# Tumor Budding, p53, and DNA Mismatch Repair Markers in Sinonasal Intestinal-Type Adenocarcinoma: A Retrospective Study Supports the Adverse Prognostic Impact of Tumor Budding

**DOI:** 10.3390/cancers16101895

**Published:** 2024-05-16

**Authors:** Sebastiano Puccio, Giuseppe Azzarello, Valeria Maffeis, Licia Laurino, Edoardo Mairani, Federica Conte, Nicola Tessari, Diego Cazzador, Elisabetta Zanoletti, Doriano Politi, Enzo Emanuelli, Giacomo Spinato, Simonetta Ausoni

**Affiliations:** 1 Section of Otorhinolaryngology—Head and Neck Surgery, Department of Neuroscience, “Azienda Ospedale Università di Padova” University of Padova, via Giustiniani, 2, 35122 Padova, Italy; sebastiano.puccio@studenti.unipd.it (S.P.); edoardo.mairani@studenti.unipd.it (E.M.); nicola.tessari@aopd.it (N.T.); diego.cazzador@unipd.it (D.C.); elisabetta.zanoletti@unipd.it (E.Z.); 2 Department of Oncology, Local Health Unit 3 Serenissima, Via don Giacobbe Sartor 4, 30035 Venice, Italy; giuseppe.azzarello@aulss3.veneto.it; 3 Anatomia Patologica, Azienda Ospedaliera Universitaria Integrata Verona (AOVR), Ospedale Borgo Trento, Piazzale Aristide Stefani, 1, 37126 Verona, Italy; valeria.maffeis@aovr.veneto.it; 4 Department of Pathology, Local Health Unit 2 Marca Trevigiana, Piazzale dell’Ospedale 1, 31100 Treviso, Italy; enzo.emanuelli@aulss2.veneto.it; 5 Department of Pathology, Local Health Unit 3 Serenissima, Via Paccagnella 11, 30174 Venice, Italy; licia.laurino@aulss3.veneto.it; 6 Department of Psychology, University of Milano-Bicocca, 20126 Milan, Italy; federica.conte@unimib.it; 7 Department of Otorhinolaryngology, Local Health Unit 3 Serenissima, Via Paccagnella, 11, 30174 Venice, Italy; doriano.politi@aulss3.veneto.it; 8 Department of Biomedical Sciences, University of Padova, Via Ugo Bassi 58b, 35121 Padova, Italy

**Keywords:** sinonasal intestinal-type adenocarcinoma, tumor budding, p53, DNA mismatch repair, microsatellite instability

## Abstract

**Simple Summary:**

This study retrospectively investigates clinicopathological characteristics and survival outcomes of patients affected by sinonasal intestinal-type adenocarcinoma with tumor budding. Tumor budding was evaluated in 32 patients and allowed the identification of high budding (>4) and low budding (≤4) groups. High-budding patients had worse overall survival, higher relapse, and disease-caused death compared to low-budding patients. On multivariate analysis, considering tumor budding, therapy, and stage as covariates, tumor budding was found to be an independent prognostic factor net of the stage of disease and the type of therapy received. Other markers, such as p53, did not show any significant prognostic correlation in sinonasal intestinal-type adenocarcinoma, whereas no change in DNA mismatch repair protein expression was detected. These results reinforce the prognostic value of tumor budding in sinonasal intestinal-type adenocarcinoma, underline the potential impact of this parameter, and encourage its use in clinical practice.

**Abstract:**

Sinonasal intestinal-type adenocarcinoma (ITAC) is a very rare, closely occupational-related tumor with strong histological similarities to colorectal cancer (CRC). In the latter, tumor budding (TB) is widely recognized as a negative prognostic parameter. The aim of this study was to evaluate the prognostic role of TB in ITAC and to correlate it with other established or emerging biomarkers of the disease, such as p53 and deficient DNA mismatch repair (MMR) system status/microsatellite instability (MSI). We retrospectively analyzed 32 consecutive specimens of patients with ITAC diagnosis treated in two institutions in Northern Italy. We reviewed surgical specimens for TB evaluation (low-intermediate/high); p53 expression and MMR proteins were evaluated via immunohistochemistry. Results were retrospectively stratified using clinical data and patients’ outcomes. According to bud counts, patients were stratified into two groups: intermediate/high budding (>4 TB) and low budding (≤4 TB). Patients with high TB (>4) have an increased risk of recurrence and death compared to those with low TB, with a median survival of 13 and 54 months, respectively. On multivariate analysis, considering TB, therapy, and stage as covariates, TB emerged as an independent prognostic factor net of the stage of disease or type of therapy received. No impact of p53 status as a biomarker of prognosis was observed and no alterations regarding MMR proteins were identified. The results of the present work provide further significant evidence on the prognostic role of TB in ITAC and underline the need for larger multicenter studies to implement the use of TB in clinical practice.

## 1. Introduction

Sinonasal intestinal-type adenocarcinoma (ITAC) is rare, accounting for 3 to 5% of malignancies of the head and neck (H&N) and 0.2 to 0.8% of all tumors [1]. In Europe, ITAC represents the most frequent malignant tumor of the sinonasal tract, with an incidence per 100,000 persons/year of 0.26 cases in men and 0.04 in women [2]. The site of origin of the tumor is commonly identified in the ethmoidal complex, but recent findings indicate that the tumor specifically originates from the olfactory cleft [3]. ITAC is defined as “intestinal” because of its histopathologic resemblance to colorectal cancer (CRC). Based on histopathological parameters, Barnes identified papillary, colonic, solid, mucinous, and mixed subtypes [4]. More recently, this classification has been revised by Kleinsasser and Schroeder [5], who proposed a subdivision into papillary-tubular cylindrical cell type (corresponding to papillary, solid, and colonic types), alveolar goblet cell type, and signed-ring cell type (corresponding to the mucinous subtype), and transitional type (corresponding to the mixed subtype).

ITAC is a highly aggressive malignancy with frequent local and metastatic spread. From a prognostic point of view, an advanced tumor stage, sphenoid sinus involvement, orbital, dural or brain infiltration, and high-grade histology are negative prognostic factors of poor outcome [6]. Unlike CRC, ITAC is etiologically associated with occupational exposure to wood dust particles. Eighty-eight percent of ITAC cases affect male leather workers, carpenters, and furniture makers [7,8,9], while the rest are sporadic tumors frequently affecting women.

Tumor budding (TB) is a morphological feature associated with adverse prognosis in many tumor types, with high representation in gastrointestinal cancers. TB is also detectable in head and neck squamous cell carcinoma (HNSCC), where it is associated with an adverse prognosis, increased risk for cervical lymph node metastases, and poor overall disease-specific and disease-free survival rates, mainly in patients with early-stage oral squamous cell carcinoma [10,11]. It has been proposed that TB in HNSCC derives from an epithelial-to-mesenchymal transition (EMT) [12], a conclusion that has been questioned by others [13] and thus remains open to debate. The emerging role of TB as a prognostic factor has recently been highlighted, as it should be considered an independent ecological island worthy of further biomolecular investigations. In other words, TB may be part of evolutionary island-like ecosystems that drive tumor cell migration and are, therefore, potential new therapeutic targets [14].

There is a great desire for new therapeutic approaches to improve the clinical management of patients affected by ITAC [13,15]. So far, only a few studies have suggested a possible prognostic role of TB in ITAC [13,16], and in all cases, no correlation has been established with other recognized biomolecular tumor markers, such as p53 [17,18].

DNA mismatch repair (MMR) system status/microsatellite instability (MSI) has growing diagnostic, prognostic, and predictive value in CRC [18]. The huge impact on clinical management potentially related to MMR deficiency and the paucity of data available in ITAC suggest further investigation of the topic.

In this retrospective study, we aimed to assess the occurrence and prognostic role of TB in an unpublished large cohort of ITAC and to investigate its possible association with p53 expression and MMR deficiency using immunohistochemistry. The results of this study provide significant evidence of the prognostic role of TB in ITACs and give insights into its wider use in real-life settings to modulate adjuvant treatment.

## 2. Materials and Methods

### 2.1. Patient Selection and Clinical Data

The analysis presented in this study was performed on formalin-fixed and paraffin-embedded specimens of ITAC patients (diagnosed consecutively between 2005 and 2023), which were retrieved from the archives of the Department of Pathology of the Hospital of Treviso (23 specimens) and the Department of Pathology of Mestre-Venezia (9 specimens). Inclusion criteria were as follows: diagnosis of CDX2- and CK20-positive intestinal-type adenocarcinoma, age > 18 years, availability of the type of treatment (surgery, radiotherapy, and surgery + chemoradiotherapy), and follow-up data. All cases were reviewed and diagnosed by expert pathologists (VM and LL) following the criteria of the 5th World Health Organization Classification of Head and Neck Tumors [19]. Moreover, both Barnes [4] and Kleinsasser and Schroeder [5] morphological classifications were applied. This study was conducted in accordance with the Declaration of Helsinki, and the protocol was approved by the Ethics Committee of Azienda ULSS 2 Marca Trevigiana (n. 421).

### 2.2. Histopathology and Immunohistochemistry

We applied the ITBCC (International Tumor Budding Consensus Conference 2016) recommendations developed for CRC [20], which were validated in 2019 [21]. Briefly, ITBCC defines TB as a single tumor cell or a cell cluster of up to 4 tumor cells at the invasive front of the tumor (peritumoral TB) or within the tumor mass (intratumoral TB), counted on hematoxylin and eosin–stained (H&E) slides in a tumor area of 0.785 mm^2^. For TB counting, the hotspot method was applied, with the aid of cytokeratin immunohistochemical staining when necessary. The hotspot was the microscopic field with the greatest number of TBs. Briefly, all the fields along the invasive front were scanned at 100× magnification before counting buds in the microscopic field with the greatest number of tumor buds at 200× magnification. The number of TBs was assessed in a field measuring 0.785 mm^2^, and the objective magnification of microscopes was normalized as previously described [20]. The absolute count of buds was registered for each case and used to classify patients with ITAC as low (0–4 buds), intermediate (5–9 buds), or high-grade budding (≥10 buds), according to the ITBCC recommendations. To create a dichotomic variable, we considered bud counts >4 as intermediate/high budding and ≤4 as low budding. In some cases, due to sample fragmentation or unreliable data on the margin of invasion, we evaluated intratumoral instead of peritumoral TB. Indeed, it has been demonstrated that intratumoral and peritumoral TB are strongly related and independently associated with a shorter survival time [22,23]. Tiny fragments obtained from resections were excluded from the analysis.

Four-micrometer-thick sections from selected samples were cut to perform immunohistochemistry. Staining was conducted automatically (DAKO, OMNIS AGILENT, Santa Clara, CA, USA), using the ENVISION FLEX Polymer detection kit (Agilent) with the commercially available antibodies listed in Table 1, and with the aid of internal controls in use in individual institutions.

Antibodies to CDX2, CK20, and CK7 were used by IHC for specimen selection. All specimens analyzed were positive for CDX2 and CK20, thus precisely confirming the diagnosis of ITAC, while CK7 was detected only in 6% of cases.

The detection of MMR status was also analyzed using antibodies specific for proteins encoded by MMR genes, namely, mutL homolog 1 (MLH1), mutS homologs 2 and 6 (MSH2 and MSH6), and postmeiotic segregation increased 2 (PMS2). MMR protein expression was interpreted as 1. maintained/positive when moderately to strong nuclear staining was present in tumor cells (with internal positive control); 2. loss, in case of complete absence of nuclear staining in tumor cells [24,25]. MMR status was considered proficient when all four proteins were expressed and deficient when at least one of the proteins was absent [26]. Table 1 shows the list of antibodies used in this study.

### 2.3. Statistical Analysis

Differences in immunohistochemical data were considered significant at *p* ≤ 0.05. Percentages were used to summarize categorical variables and means, and standard deviations were used for continuous variables. Descriptive statistics for patient and tumor characteristics, treatment and follow-up, and MMR, p53. and TB are presented.

First, univariable analyses were performed. Fisher’s exact test was used to assess the effects of p53 and TB on death by disease and relapse. A bootstrapped t-test was used to examine differences in overall survival (months elapsed from diagnosis to death) between low and high TB groups, accompanied by a Kaplan–Meier curve. Finally, Kendall’s bivariate correlations between overall survival and TB, as well as stage and therapy, were examined.

A multiple regression analysis was performed considering survival (in months) as the dependent variable and TB, stage, and therapy as the independent variables to determine whether TB held a specific prognostic value. Analyses were performed in R using the functions boot.t.test from package MKinfer for the bootstrapped t-test and survfit from package ggsurvfit to draw the Kaplan–Meier curve. The software used for graphics was Prism7.

## 3. Results

### 3.1. Patients and Tumor Characteristics

**Figure 1:**
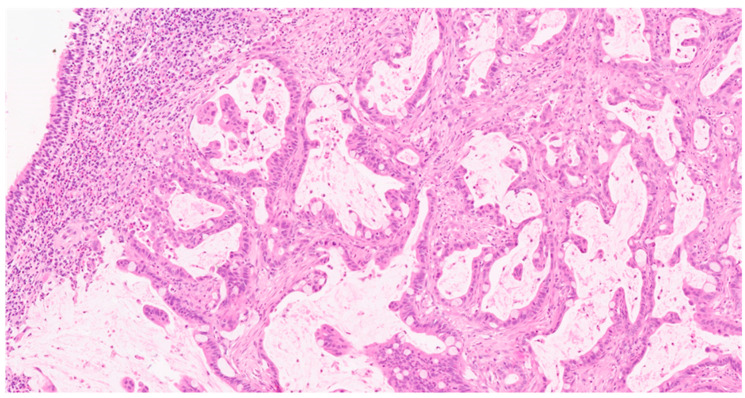
Intestinal-type adenocarcinoma of the sinonasal tract with tubulopapillary architecture and goblet cells (H&E, 100× magnification).

Thirty-four patients were enrolled in this study. Two patients from whom TB could not be determined were excluded from the analysis (a very tiny fragment of tumor without enough stroma in one case and excessive fragmentation of the specimen in the other case), resulting in a final cohort of 32 patients. Table 2 summarizes the clinical and pathological variables of this cohort and their correlation with TB. The mean age of patients was 67 ± 11 years (range 47–88). Seventy-eight percent of patients (*n* = 25) were male, and 60% had a history of occupational exposure to either wood (44%, *n* = 14) or leather (16%, *n* = 5). The most represented histopathological subtype was the colonic (23 patients, 72%), followed by the mixed subtype in seven (22%) samples and the mucinous in only two (6%). Tumor grading was as follows: G1 in 7 patients (22%), G2 in 21 patients (66%), and G3 in 4 patients (12%). Pathologic tumor stage was distributed as follows: stage I in 5 (16%) cases, stage II in 11 (34%), stage III in 5 (16%), and stage IV in 10 (31%). For one patient (3%), the tumor stage could not be determined. Figure 1 shows a representative ITAC.

### 3.2. Patient Treatment and Follow-Up

Patients were treated with different modalities. Thirteen out of thirty-two (41%) patients with early-stage tumors received only surgery. Sixteen out of thirty-two (50%) patients underwent surgery and adjuvant radiotherapy or chemoradiotherapy. Only 3/32 (9%) patients received either radiotherapy or chemoradiotherapy; therefore, TB was evaluated on bioptic material. Surgical margins were positive in only 4/32 (12%) cases. All patients with positive margins (R1) received adjuvant radiotherapy.

The mean follow-up time was 43 months; 18 patients had a follow-up time of less than or equal to 24 months. During the follow-up, 11 (34%) patients had a relapse. At the last follow-up, 21 (66%) patients were alive without disease, 1 (3%) patient was alive with disease, 9 (28%) patients died from disease, mainly from local recurrence, and only 1 (3%) patient died from other causes.

### 3.3. TB Evaluation, MMR Status, and p53 Analysis

**Figure 2:**
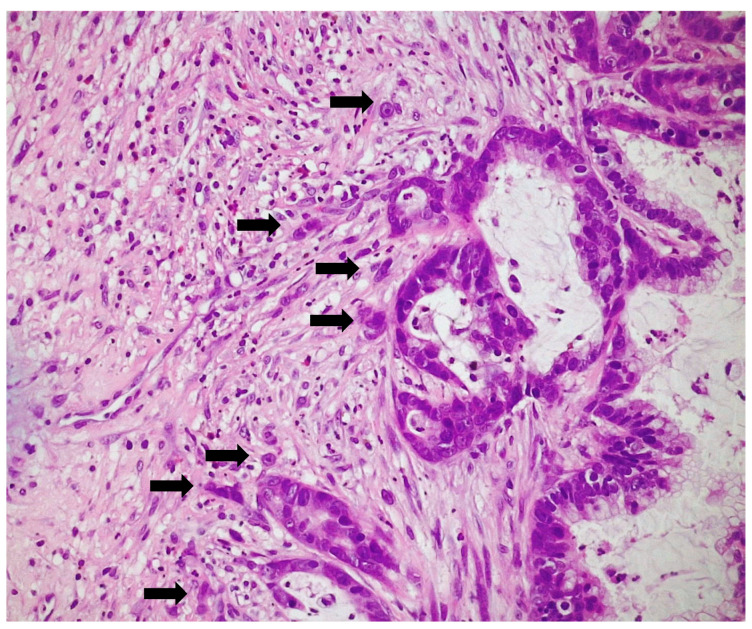
Tumor budding at the invasive front of the tumor (arrows) (H&E, 200× magnification).

TB was assessed. Out of a total of 26 patients in the “low” budding group, 17 specimens (53%) had a TB value of 0, while 9 specimens (28%) had a TB value of ≤4. In the “high” budding group, six specimens (19%) had a TB value of >4. TB could not be determined in two specimens (6%). Figure 2 illustrates a representative TB in ITAC. The association between TB and clinical and pathological variables is outlined in Table 2. TB values ranged from 0 to a maximum of 18.

**Figure 3:**
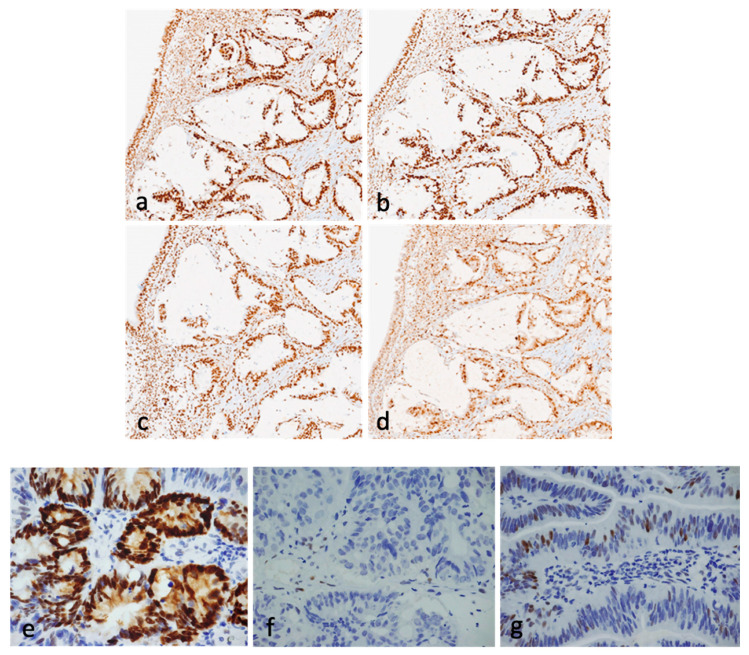
MMR protein nuclear positivity and p53 expression in ITAC. MMR protein nuclear positivity is shown for MSH2 (**a**), MSH6 (**b**), MLH1 (**c**), and PMS2 (**d**). p53 Detection is shown in three different conditions corresponding to overexpression (**e**), null (**f**), and wild-type pattern (**g**).

MMR proteins MLH1, PMS2, MSH2, and MSH6 were expressed in all specimens (Figure 3a,b,c, and d, respectively), thus supporting microsatellite stability (MSS).

p53 Expression was also investigated via immunohistochemistry, based on the evidence that in most cases, *TP53* status can be surrogated through three different p53 protein expression patterns, i.e., mutant pattern (overexpression or null phenotype) and wild-type pattern [27]. p53 Overexpression corresponds to strong nuclear positivity involving at least 80% of the tumor cells, null staining to absent nuclear detection of p53, and wild type to a mixture of nuclear negative and weakly to strongly positive nuclei. From the molecular point of view, p53 overexpression accounts for in-frame TP53 mutations in the DNA binding domain, null for disrupted-type TP53 mutations, and wild type for the absence of mutations. In 16 patients (50%), p53 was found to be either overexpressed (14 patients, 44%) (Figure 3e) or null (2 patients, 6%) (Figure 3f) compared to internal controls (Figure 3g). Expression of immunohistochemical markers in the specimens is summarized in Table 3.

### 3.4. Univariable and Multivariable Analyses

Fisher’s exact tests indicated that both deaths due to the index disease and relapse were independent of p53 expression: the death rate was 27% vs. 25% in patients with normal vs. overexpressed or null p53 (*n* = 31, *p* = 1.000); the relapse rate was 27% vs. 31% (*n* = 31, *p* = 1.000). On univariate analysis, considering death and relapse as dependent variables, p53 did not prove to be a statistically significant prognostic factor (*p* = 0.76 and 0.46, respectively).

**Figure 4:**
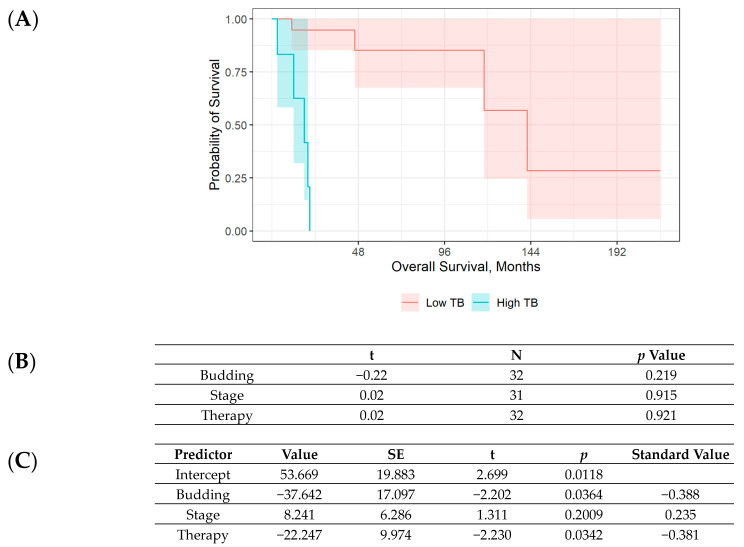
(**A**) Plot of Kaplan–Maier curve showing overall survival of patients with low TB and high TB. (**B**) Kendall’s bivariate correlations between overall survival and TB, tumor stage, and therapy. (**C**) Multiple regression analysis of patients with respect to TB, tumor stage, and therapy.

A similar test showed that, compared to the low-budding group, patients with high budding had a significantly higher rate of death due to illness (15% vs. 83%, *n* = 32, *p* = 0.003). Relapse rate and overall survival were nominally worse (i.e., 23% vs. 67% and 50 vs. 14 months, respectively) but not significantly so Fisher’s exact test was used to analyze relapse (*n* = 32, *p* = 0.060), and a bootstrapped t-test was used to analyze overall survival (bootstrapped difference of means = 35.41, SE = 21.70, 95% CI = −13.49; 74.10) (Figure 4A). Kendall’s bivariate correlations (Figure 4B) did not detect any significant associations between either TB, tumor stage, or therapy and overall survival (between τ = −0.22 and τ = 0.02, *p* ≥ 0.219). However, in the multiple regression considering TB, therapy, and tumor stage as predictors of overall survival, TB was found to be an independent prognostic factor net of the stage of disease or the type of therapy received (β = −0.388, *p* = 0.036) (Figure 4C).

## 4. Discussion

In this study, we demonstrate that TB is an independent prognostic marker in terms of overall survival (OS) and risk of recurrence in ITAC patients. We also show, for the first time, that TB is an independent prognostic factor for OS, net not only of the stage but also of the therapies implemented for the patients. Furthermore, in agreement with previous observations [18,28], we show that p53 has no prognostic impact in ITAC and that MMR protein expression is conserved, thus confirming a microsatellite-stable status (MSS) in these tumors.

The primary endpoint of our study was to establish whether TB may represent a prognostic marker of ITAC. We were able to establish the statistical relevance of TB detection by stratifying patients into two groups: those with intermediate/high-grade TB versus those with low-grade TB. Clinically, patients with high-grade TB (>4 tumor buds) had an increased risk of recurrence (*p* = 0.06) and death (*p* = 0.003) compared to those with low TB (≤4 tumor buds) (median survival of 13 and 54 months, respectively) in our cohort, as previously found in CRC [29].

The multivariate analysis, which considered stage, budding, and therapy, confirmed the correlation between TB and OS. Ultimately, budding was confirmed as an adverse prognostic factor since patients with intermediate/high budding had both worse OS and a greater risk of recurrence (34% of patients).

To the best of our knowledge, TB in ITAC has been so far investigated only in two other studies, with a number of enrolled patients comparable to ours. The first contribution by Maffeis et al. (32 patients) was, unlike ours, monocentric and compared negative versus positive TB cases (despite evaluation and stratification being conducted using ITBCC recommendations). Similar to CRC, the authors found an association between the presence of TB and some pathological and clinical parameters, like the presence of lymphovascular invasion, recurrence, and death from the disease [13]. The second study by Meerwein et al. (31 patients) was also monocentric and applied the same TB stratification. The authors concluded that TB is an independent negative prognostic marker regardless of the tumor subtype and stage [16]. Our data strengthen the evidence of an independent prognostic value of TB in ITAC and are therefore in line with previous conclusions. Moreover, evidence in the literature suggests that the prognostic role of TB is independent of the assessment method [23,30,31]. However, the use of a method that has international consensus allows comparison between studies. Net of the incidence of this rare neoplastic disease, our retrospective study retains some limitations, such as (1) the low number of patients; (2) TB evaluation on bioptic material in three patients who did not undergo surgery; (3) the challenge imposed by en-bloc resection.

In a parallel analysis, we investigated p53 expression and MMR status in ITAC via immunohistochemistry. Eighteen patients (54%), equally distributed throughout tumor stages I–IV, had a p53 mutant pattern. In contrast to TB, however, no impact of p53 as a prognostic biomarker was observed. TP53 is one of the most frequently mutated genes in HNSCCs (up to 85%) [17,32,33] and correlates with relapse and chemoresistance and ultimately with prognosis [17,34]. A high frequency of mutated TP53 (over 70%) has also been demonstrated in a large collection of sinonasal cancers due to occupational exposure [35].

Through a retrospective PCR-based analysis in ITACs, Licitra et al. [36] and Bossi et al. [18] concluded that wild-type TP53 is predictive of response to primary chemotherapy. Specifically, a functional p53 may predict cisplatin-based chemotherapy efficacy and affect prognosis, but there is no impact of p53 functional status in treatment-naïve patients submitted to surgery and radiotherapy. These findings, together with results produced by other authors [28] and with our results based on immunohistochemistry, strongly support a predictive rather than a prognostic role of p53 functional status in ITAC. Although a simplified vision is important to orient the clinical practice, other studies appear necessary to resolve the complexity of p53. The presence of TP53 mutation does not necessarily imply p53 inactivation, and immunohistochemistry does not provide quantitative results, nor does it establish p53 residual protein function [27,37]. In addition, the heterogeneity of TP53 mutations in different tumors, including head and neck cancers, impacts equally variable prognosis profiles [38,39,40,41,42].

We also considered MMR protein expression in ITAC as a surrogate of MSI and found a normal profile of nuclear expression in our material. This is in contrast with colorectal cancer, where microsatellite instability (MSI) is present in 15% of non-metastatic disease and in 5% of the metastatic setting [29] and confirmed peculiar molecular characteristics of ITAC. Previous work in experimental models and tumor cell lines suggested that an MMR-deficient profile could be a predictive factor for a poor response to chemotherapeutics, i.e., cisplatin, carboplatin, and methylating agents, at variance with the efficacy of immunotherapy in CRC with MMR/MSI deficiency [43]. Our data are in line with previous studies. Martinez et al. found MSI via PCR in 1/41 ITACs and 5/24 HNSCCs and concluded that this mechanism does not play an important role in ITAC tumorigenesis but may be relevant in HNSCCs [44]. We conclude that MSI is not involved in the pathogenesis of ITAC and, consequently, cannot be included as a prognostic variable.

## 5. Conclusions

This is the third study that investigated the prognostic role of TB in patients affected by ITAC, and the first study that explored the potential TB association with p53 expression and MMR status in this disease. So far, only two studies (for a total of 63 patients) have investigated the presence of TB in ITAC. The authors found that TB is a negative prognostic marker, similar to CRC and many HNSCCs [13,16], but their results required further validation.

At variance with previous contributions, we provide (1) a multicentric study, which included previously unpublished cases of this rare tumor; (2) evidence of an independent negative prognostic role of TB, net not only of the stage of the disease but also of therapy; (3) a larger study group on MMR status in ITAC. On multivariate analysis, considering tumor budding, therapy, and stage as covariates, TB was found to be an independent prognostic factor net of the stage of disease and the type of therapy received. Other markers, such as p53 and MMR status, failed to show any significant correlation with ITAC. These results reinforce the prognostic value of TB in ITAC and underline the potential impact of this investigation to encourage its use in clinical practice.

How could these data help to choose the best therapeutic strategy, and where can they fit into a hypothetical flow diagram in light of current knowledge on prognostic and/or predictive factors? Clinical data confirm that major prognostic factors of ITAC are (1) positive resection margins and (2) tumor grade of differentiation [45,46,47]. In these cases, radiotherapy and chemotherapy should be considered postoperatively as adjuvant treatments. Despite advances in these approaches, the overall prognosis, in terms of 5-year OS, is poor, ranging from 80% in stage I to 30% in stage IV. Our data on the prognostic value of TB, independent not only of the stage but also of the therapy used, may allow further stratification of patients eligible for innovative therapies in controlled clinical trials. There is growing evidence of different targetable signaling pathways, among which the most promising appears to be the mTOR signaling and the ERK/MAPK pathway [48,49]. mTOR and ERK pathways are activated in a large proportion of ITAC cases. Inhibitors of these two pathways in the ITAC-3 cell line have shown a cytostatic effect and growth inhibition [50].

## Figures and Tables

**Table 1:** Antibodies used for the immunohistochemical analysis.

Antigen	Clone	Source	Company
CDX2	DAK-CDX2	Mouse	Agilent Dako
CK7	OV-TL 12/30	Mouse	Agilent Dako
CK20	KS 20.8	Mouse	Agilent Dako
P53	DO-7	Mouse	Agilent Dako
PMS2	EP52	Mouse	Agilent Dako
MLH1	ES05	Mouse	Agilent Dako
MSH2	FE11	Mouse	Agilent Dako
MSH6	EP49	Mouse	Agilent Dako

**Table 2:** Associations between tumor budding (TB) and clinical and pathological variables. LVI, lymphovascular invasion; Exposure, exposure to either wood or leather; *p*-value < 0.05 is considered statistically significant.

	N = 32	TB > 4	TB ≤ 4	*p* Value
**Mean age**	32	67.8	66.7	>0.05
**Sex** Male Female	25 7	5 1	20 6	>0.05
**Disease stage** I II III IV	5 11 5 10	1 0 1 4	5 11 4 6	>0.05
**ITAC subtype** Colonic Mixed Mucinous	23 7 2	2 3 1	19 6 1	>0.05
**Grading** 1 2 3	7 21 4	0 5 1	7 16 3	>0.05
**LVI** Present Absent	5 27	2 4	3 23	>0.05
**Exposure (n = 20)** Present Absent	19 1	3 0	16 1	>0.05
**Recurrence** Present Absent	11 21	5 1	6 20	0.01
**Dead of disease** No evidence of disease	9 22	5 1	4 22	0.003

**Table 3:** MMR status and p53 expression in the specimens analyzed. MSH2, MutS Homolog 2; MSH6, MutS Homolog 6; PMS2, postmeiotic segregation increased; MLH1, mutL homolog 1.

Protein	N = 32
MSH,2 MSH6, PMS2, MLH1	0
p53	
Null	2 (6%)
Overexpressed	14 (44%)
Wild-type (control)	16 (50%)

## Data Availability

According to the local ethics committee, data will be available upon request to Giacomo Spinato (giacomo.spinato@unipd.it).
